# Enhanced screening for posttraumatic stress disorder and comorbid diagnoses in children and adolescents

**DOI:** 10.3402/ejpt.v6.26661

**Published:** 2015-08-28

**Authors:** Eva Verlinden, Brent C. Opmeer, Els P. M. Van Meijel, Renée Beer, Carlijn De Roos, Iva A. E. Bicanic, Francien Lamers-Winkelman, Miranda Olff, Frits Boer, Ramón J. L. Lindauer

**Affiliations:** 1 Department of Child and Adolescent Psychiatry, Academic Medical Center, University of Amsterdam, Amsterdam, The Netherlands; 2 De Bascule, Academic Center for Child and Adolescent Psychiatry, Amsterdam, The Netherlands; 3 Clinical Research Unit, Academic Medical Center, University of Amsterdam, Amsterdam, The Netherlands; 4 GGZ Rivierduinen, Psychotraumacenter for Children and Youth, Leiden, The Netherlands; 5 National Psychotraumacenter for Children and Youth, University Medical Center Utrecht, Utrecht, The Netherlands; 6 Children's and Youth Trauma Center, Haarlem, The Netherlands; 7 Department of Psychiatry, Academic Medical Center, University of Amsterdam, Amsterdam, The Netherlands

**Keywords:** PTSD, trauma, diagnosis, screening, assessment, children, adolescents, CRIES

## Abstract

**Background:**

Posttraumatic stress disorder (PTSD) can be a debilitating disorder and often co-occurs with other psychiatric disorders, such as mood, behavioral, and anxiety disorders. Early identification of PTSD and psychiatric comorbidity is highly relevant in order to offer children appropriate and timely treatment. The Children's Revised Impact of Event Scale (CRIES-13) is a reliable and valid self-report measure designed to screen children for PTSD. However, this measure is not useful as a screen for psychiatric comorbidity in children with probable PTSD.

**Objective:**

This study evaluated the screening accuracy of the CRIES-Plus, that is, the CRIES-13 combined with 12 additional items to detect psychiatric comorbidity.

**Method:**

The CRIES-Plus was completed by 398 Dutch children (7–18 years) exposed to various traumatic events. Psychiatric diagnoses were assessed using the Anxiety Disorders Interview Schedule for DSM-IV: Child version.

**Results:**

Six additional items were significantly associated with mood disorders, three items were associated with behavioral disorders, and five items with anxiety disorders. Additional items associated with mood and anxiety disorders demonstrated good discriminatory ability, with cut-off scores of ≥14 and ≥10, respectively. Items associated with behavioral disorders had poor to fair discriminatory ability, with no clear cut-off point.

**Conclusions:**

Our findings support the use of the CRIES-Plus to screen for PTSD and comorbid disorders which may help clinicians in assigning appropriate follow-up diagnostic and clinical care.

Worldwide, children are exposed to traumatic life events, such as natural disasters, school violence, car accidents, sexual assault, or child maltreatment. In fact, more than two-third of children in community samples report having experienced a traumatic life event by age 16 (American Psychological Association, 2008). However, exposure prevalence estimates among children and adolescent vary considerably, depending on several factors including the sample type, informant source, and the type of instrument (Fairbank & Fairbank, 2009).

In the aftermath of a traumatic life event, nearly all children and adolescents express some kind of psychological distress or behavioral change in their acute phase of recovery (American Psychological Association, 2008). For example, it is very common for children to have some nightmares, sleeping problems, and angry outbursts, or to feel irritable or more anxious after experiencing a traumatic life event. These reactions are often referred to as normal stress reactions to abnormal events. For most children, these responses are short-term and will gradually diminish over time. However, a significant minority of children will develop long-lasting difficulties that may affect their overall daily functioning (Fairbank & Fairbank, 2009; Gabbay, Oatis, Silva, & Hirsch, 2004).

Posttraumatic stress disorder (PTSD) is probably the most commonly studied and the most frequent and devastating disorder that may occur after a traumatic life event (Galea, Nandi, & Vlahov, 2005). Exposure to traumatic events, however, may lead to significant levels of other comorbid psychiatric disorders. The most common comorbid disorders found to exist with PTSD in children include mood disorders, behavioral disorders, and anxiety disorders (Copeland, Keeler, Angold, & Costello, 2007; Famularo, Fenton, Kinscherff, & Augustyn, 1996; Hubbard, Realmuto, Northwood, & Masten, 1995). Knowledge and understanding of comorbidity is highly relevant from a diagnostic and therapeutic point of view as comorbidity may complicate the diagnostic process and influence the course, prognosis, and treatment of children (Burgic-Radmanovic & Burgic, 2010).

Without proper treatment, PTSD and comorbid psychiatric disorders can persist for years and significantly interfere with children's psychosocial and cognitive development (Yule, Bruggencate, & Joseph, 1994). Therefore, assessment of PTSD and psychiatric comorbidity is highly relevant in order to prevent chronic health problems and to offer these children appropriate and timely treatment if needed.

Structured diagnostic interviews are the gold standard in the assessment of PTSD and psychiatric comorbid disorders. However, in-depth interviews are time-consuming and often require professional training (Stallard, Velleman, & Baldwin, 1999; Verlinden et al., 2014b). Alternatively, screening tools may serve as a first step to identify children who may need further assessment and/or treatment. One of the most widely used screening tools for PTSD in children is the Children's Revised Impact of Event Scale (CRIES; Children and War Foundation, 1998). The CRIES appears to be a reliable and valid measure to screen for posttraumatic stress in children and adolescents (Dow et al., 2012; Perrin, Meiser-Stedman, & Smith, 2005; Verlinden et al., 2014b). However, to be clinically useful, a trauma screen should assess a broad range of symptoms, especially those of the most common comorbid conditions, depression and anxiety disorders (Stallard et al., 1999). It has been suggested that adding relevant items to the CRIES may serve this purpose (Perrin et al., 2005), but to our knowledge this has not yet been investigated. Against this background, the purpose of this study was to evaluate the screening accuracy of the CRIES-Plus, that is, the Children's Revised Impact of Event Scale (CRIES-13) in combination with several additional items, to detect PTSD and psychiatric comorbidity. Reliability and predictive ability (i.e., sensitivity, specificity, and positive/negative predictive value [PPV/NPV]) of the CRIES-Plus were evaluated. The additional items were created to assess the most common comorbid disorders, that is, mood disorders, behavioral disorders, and anxiety disorders. The study includes a large sample of Dutch children exposed to a wide variety of traumatic events and with a diversity of comorbid psychiatric disorders.

## Method

### Participants

The study population consisted of a clinically referred sample of 398 children and adolescents (referred to as “children” in this manuscript) aged between 7 and 18 years. The sample had slightly more girls (*N*=249, 62.6%) than boys with an average age of 12.80 years (SD=3.01). Children were exposed to various types of traumatic events. Table 1 shows the type of events mentioned by the children as worst event or the event they found most distressing. Children were recruited from the department of youth welfare and four specialized child trauma centers in different regions in the Netherlands. Data were collected between June 2008 and March 2011.

**Table 1:** Type of worst traumatic event mentioned by the child

Type of event	*N*	%
Sexual assault	132	32.9
Domestic violence^a^	106	26.7
Accident or injury^b^	47	11.8
Physical violence	34	8.5
Traumatic grief	25	6.3
Bullying	21	5.3
Emotional abuse or neglect	7	1.8
Serious illness or death of a loved one	6	1.5
Serious illness of child	5	1.3
Stalking	3	0.8
Out of home placement	3	0.8
Other events^c^	9	2.3

*N*=398.

a Any incident of threatening behavior, violence, or abuse (psychological, physical, sexual, economic, or emotional) between adults who are or have been intimate partners or family members;

b single events such as traffic accidents, falls, robberies, shooting incidents;

c war exposure, victim of lover boys, stay in asylum center, stay in (women's) shelter, parent in prison.

### Measures

#### The Children's Revised Impact of Event Scale

The CRIES-13 (Children and War Foundation, 1998; Olff, 2005) is a brief, self-report questionnaire designed to screen for PTSD in children aged 8 years and older. It consists of four questions to assess intrusion, four questions to assess avoidance, and five questions to assess arousal. Children rate the frequency with which they have experienced each of the items during the past week using a four-point Likert-scale (0=*not at all*, 1=*rarely*, 3=*sometimes*, 5=*often*). The CRIES-13 has been successfully used in a number of studies with children aged 7 years and above (Children and War Foundation, 1998; Verlinden et al., 2013, Verlinden et al., 2014b). Psychometric properties have been previously reported (Giannopoulou et al., 2006; Perrin et al., 2005; Smith, Perrin, Dyregrov, & Yule, 2003; Verlinden et al., 2014b), showing the CRIES-13 to be a valid measure of posttraumatic stress. In this study, the internal consistency of the CRIES-13 was *α*=0.89.

#### Additional items

The additional questions were created by a team of five experts including a clinical psychologist, a psychotherapist, a child psychiatrist, and a professor in child maltreatment. All team members had extensive experience in the trauma field, both clinically and in research. Additional items were based on other well-validated questionnaires, that is, the Trauma Symptom Checklist for Young Children (Briere, 1996, 2005), the Strengths and Difficulties Questionnaire (Goodman, 1997), the Child Behavior Checklist (Achenbach & Rescorla, 2001), the Revised Child Anxiety and Depression Scale (Chorpita, Yim, Moffitt, Umemoto, & Francis, 2000), and the Children's Depression Inventory (Kovacs, 1992). The team members formulated 12 new items related to mood, behavioral, and anxiety disorders. Mood disorders include depression and dysthymia; behavioral disorders include Attention Deficit Hyperactivity Disorder (ADHD); anxiety disorders include separation anxiety disorder (SAD), social phobia, specific phobia, generalized anxiety disorder (GAD), obsessive compulsive disorder (OCD), panic disorder (PD), and agoraphobia. Consensus was reached through collaborative discussions. The final items are shown in Table 2. Similar to the original CRIES-13, children rate the frequency with which they have experienced each of the items during the past week using a four-point Likert-scale (0=*not at all*, 1=*rarely*, 3=*sometimes*, 5=*often*). Items 5, 7, 10 and 11 are to be reverse scored.

**Table 2:** Additional items

Item
1. Is there anything you're afraid of?
2. Are you afraid you might do or think something bad?
3. Are you worried?
4. Do you suffer from tension in your arms, legs or neck?
5. Do you normally do what people ask?
6. Do you get accused of lying and cheating?
7. Do you think before you do something?
8. Do you get into arguments?
9. Are you unhappy or down in the dumps?
10. Do you enjoy doing things?
11. Do you think things are going okay with you?
12. Do you feel like crying?

#### The Anxiety Disorders Interview Schedule for DSM-IV

The Anxiety Disorders Interview Schedule for DSM-IV: Child and Parent Version (ADIS-C/P; Siebelink & Treffers, 2001; Silverman & Albano, 1996) is a semi-structured interview for diagnosing anxiety disorders in children and adolescents aged 7–17 years. In addition, other related disorders such as mood and externalizing disorders are included to assess a full diagnostic picture. Symptoms are rated by respondents as either present (“yes”), absent (“no”), or “other” (e.g., “sometimes” or “do not know”). Furthermore, impairment ratings of each diagnosis are considered using a nine-point Likert-like scale (0–8). Diagnoses were based upon a current time frame according to DSM-IV requirements. An extended adaptation of the PTSD module was used to assess a complete trauma history (Verlinden, Van Meijel, & Lindauer, 2009). The ADIS-C/P appears to be a reliable instrument for deriving DSM-IV anxiety disorder symptoms and diagnoses in children and adolescents with good to excellent test–retest and inter-rater reliability (Lyneham, Abbott, & Rapee, 2007; Silverman, Saavedra, & Pina, 2001). For the current study, the inter-rater agreement was excellent (*κ*=0.89).

### Procedure

Prior to the study, approval was obtained from the Medical Ethical Committee of the Academic Medical Center in Amsterdam. Children and parents were invited to participate in the study after comprehensive explanation. Children with evidence of mental retardation and children reporting psychotic symptoms were excluded. Written informed consent was obtained from children aged 12 years and above and from all parents. Children completed an assessment involving the CRIES-Plus, that is, the CRIES-13 in combination with the additional items, along with a child diagnostic interview (ADIS-C) to determine psychiatric diagnoses. Assessments were conducted by five psychologists, all with one or more years of experience in the trauma field and familiar with psychodiagnostic assessment of children. The psychologists were trained extensively in the assessment of the CRIES-Plus and the ADIS-C. Children completed the CRIES-Plus by themselves and were instructed to base their posttraumatic symptom reports upon the worst event or the event considered most distressing. A psychologist was present to answer any questions when necessary. Thereafter, the child was interviewed by the psychologist. All interviewers were instructed to follow the introductions and questions of the interview literally, to prevent any interviewer bias. Only in case of an unclear answer, the interviewer could ask for some clarification. In addition, there were regularly scheduled meetings for all research members to discuss all difficulties with each other. In any doubt, a child psychiatrist with extensive experience in the trauma field was consulted.

### Statistical analyses

For the CRIES-Plus, data were counted as missing if more than one item on a subscale (>25.0%) was left blank. Of the 398 children, six were excluded due to missing data on the CRIES-Plus, leaving a total of 392 cases for further analysis. Four children had only one missing item on a subscale; these items were scored zero (Smith, Perrin, Yule, Hacam, & Stuvland, 2002). Score differences on the CRIES-Plus between children with and without PTSD were evaluated with the independent-sample *t*-test or Chi-square test. The correlation between the total scale of the CRIES-13 and all of the additional items was quantified with the Pearson correlation coefficient. The internal consistency of the additional items was assessed using Cronbach's α.

Simple logistic regression analyses were performed to identify additional items significantly associated with PTSD, mood disorders, behavioral disorders, and anxiety disorders. Multiple logistic regression analyses were performed using stepwise backward procedures with 0.10 alpha levels of removal to identify a combination of additional items significantly associated with PTSD, mood disorders, behavioral disorders, and anxiety disorders. Model selection was based on likelihood ratio test statistics. Fit of the final model was assessed using Nagelkerke R-square. The predictive value of the final model was assessed by receiver-operating characteristics (ROC) curve. Sensitivity (the proportion of children correctly identified as having a disorder), specificity (the proportion of children correctly identified as not having a disorder), PPV (the proportion of children who screen positive on the additional items and actually have a disorder), NPV (the proportion of children who screen negative on the additional items and do not have a disorder), and overall efficiency (the proportion of children correctly identified by the additional items) were calculated. Cut-off scores were chosen to strike the best balance between high sensitivity and reasonable specificity. Statistical Package for Social Sciences (SPSS) version 19.0 for Windows was used to perform all statistical analyses.

## Results

According to the ADIS-C, 178 children (45.4%) met DSM-IV diagnostic criteria for PTSD. Mean scores and standard deviations for the total scale of the CRIES-13 and the additional items are shown in Table 3. Children with PTSD had significantly higher scores on the total scale of the CRIES-13 and almost all of the additional items compared to children without PTSD (Table 3). A positive correlation was found between the CRIES-13 and all of the additional items (*r*=0.75, *p*<0.001). Cronbach's α for internal consistency of the additional items was *α*=0.76.

**Table 3:** Mean and standard deviation for the CRIES-13 and the additional items

	PTSD (*n* = 178)		No PTSD (*n* = 214)		Total (*N* = 392)	
			
Scale	Mean	SD	Mean	SD	Mean	SD
CRIES-13 total score	42.48	10.47	19.48	13.06	29.92	16.55
Additional items						
1	2.80	1.54	1.45	1.58	2.06	1.70
2	2.38	1.78	0.87	1.40	1.56	1.75
3	3.30	1.66	1.34	1.63	2.23	1.91
4	2.16	1.96	0.86	1.46	1.45	1.82
5	0.88	1.20	1.00	1.46	0.95	1.34
6	1.34	1.58	0.89	1.47	1.09	1.54
7	1.20	1.47	1.17	1.49	1.18	1.48
8	2.09	1.72	1.73	1.45	1.89	1.59
9	2.74	1.79	1.02	1.49	1.80	1.84
10	0.96	1.21	0.53	1.20	0.73	1.22
11	1.49	1.25	0.88	1.26	1.16	1.29
12	2.99	1.78	1.24	1.47	2.04	1.84
Total score	24.32	8.61	13.00	8.56	18.14	10.26

Groups were based on the Anxiety Disorders Interview Schedule for DSM-IV: Child Version (ADIS-C). Independent-sample *t*-test was conducted to examine differences between children with and without PTSD. All *p*-values were <0.05 except for items 5 and 7. CRIES = Children's Revised Impact of Event Scale; PTSD = posttraumatic stress disorder.

Table 4 reports frequency rates of all (comorbid) psychiatric diagnoses according to the ADIS-C. Of all children, 41.1% had at least one (comorbid) psychiatric disorder. Prevalence rates indicate that specific phobia was the most prevalent disorder (19.1%) followed by GAD (15.6%). As can be seen in Table 4, children with PTSD were more likely to show (comorbid) psychiatric disorders than were children without PTSD. Furthermore, the number of (comorbid) disorders were significantly different between children with and without PTSD, χ^2^ (4, *N*=392)=103.56, *p*<0.001.

**Table 4:** Comorbid psychiatric diagnoses

	Total (*N=*392)		PTSD (*n=*178)		No PTSD (*n=*214)	
			
Variable	*n*	%	*n*	%	*n*	%
Any disorder	161	41.1	121	68.0	40	18.7
Mood disorder^a^	55	14.0	49	27.5	6	2.8
Behavioral disorder^b^	40	10.2	28	15.7	12	5.6
Anxiety disorders						
SAD	20	5.1	16	9.0	4	1.9
Social phobia	39	9.9	30	16.9	9	4.2
Specific phobia	75	19.1	55	30.9	20	9.3
GAD	61	15.6	54	30.3	7	3.3
OCD	14	3.6	13	7.3	1	0.5
PD and/or agoraphobia	16	4.1	14	7.9	2	0.9
No. of comorbid disorders						
0	231	58.9	57	32.0	174	81.3
1	75	19.1	50	28.1	25	11.7
2	43	11.0	33	18.5	10	4.7
3	22	5.6	18	10.1	4	1.9
≥4	21	5.4	20	11.2	1	0.5

Groups were based on the Anxiety Disorders Interview Schedule for DSM-IV: Child Version (ADIS-C). Pearson chi-square analysis was conducted to examine differences between children with and without PTSD. All *p*-values were ≤0.001. SAD=separation anxiety disorder; GAD=generalized anxiety disorder; OCD=obsessive compulsive disorder; PD=panic disorder; PTSD=posttraumatic stress disorder.

a Mood disorder includes depression and dysthymia;

b behavioral disorder includes attention deficit hyperactivity disorder (ADHD).

Results of simple and multiple logistic regression analyses to assess the association between individual variables as well as a combination of variables with PTSD, mood disorders, behavioral disorders, and anxiety disorders, are presented in Table 5. None of the additional items significantly improved the prediction of PTSD when added to the total scale of the CRIES-13 (order of exclusion; items 1, 2, 6, 7, 9, 10, 8, 12, 5, 4, 3, and 11). Nagelkerke R-square for this final model was 0.61. Six out of 12 additional items (items 1, 3, 4, 7, 9, and 12) were significantly associated with mood disorders (order of exclusion; items 5, 6, 11, 2, 10, and 8). Nagelkerke R-square for this final model was 0.43. Three additional items (items 6, 8, and 9) were significantly associated with behavioral disorder (order of exclusion; items 3, 4, 2, 11, 12, 1, 5, 7, and 10). However, these items explained only 12% of the variance (Nagelkerke R-square=0.12). Five out of 12 additional items (items 1, 3, 8, 9, and 11) were significantly associated with anxiety disorders (order of exclusion; items 6, 5, 12, 7, 10, 4, and 2). Nagelkerke R-square for this final model was 0.44.

**Table 5:** Association between individual variables as well as a combination of variables with PTSD, mood disorders, behavioral disorders, and anxiety disorders (odds ratio)

	PTSD		Mood		Behavioral		Anxiety	
				
Variable	Simple	Multiple	Simple	Multiple	Simple	Multiple	Simple	Multiple
CRIES-13 total scale	1.16**	1.16**						
Item 1 (anxious)	1.68**	–	1.27**	0.80*	1.05	–	1.76**	1.45**
Item 2 (bad thinking)	1.75**	–	1.49**	–	1.06	–	1.67**	–
Item 3 (worry)	1.88**	–	1.91**	1.42**	1.21**	–	1.84**	1.46**
Item 4 (physical tension)	1.53**	–	1.57**	1.29**	1.21**	–	1.33**	–
Item 5 (obey)	0.93	–	1.00	–	0.93	–	0.94	–
Item 6 (lying/cheating)	1.21**	–	1.21**	–	1.42**	1.33**	1.16**	–
Item 7 (think before acting)	1.01	–	1.23**	1.29**	1.24**	–	1.01	–
Item 8 (arguing)	1.16**	–	1.34**	–	1.37**	1.23*	1.33**	1.24**
Item 9 (unhappy)	1.78**	–	1.99**	1.47**	1.30**	1.20*	1.71**	1.18*
Item 10 (enjoy)	1.35**	–	1.60**	–	1.01	–	1.50**	–
Item 11 (things going okay)	1.47**	–	1.41**		1.22*	–	1.70**	1.43**
Item 12 (crying)	1.82**	–	1.95**	1.41**	1.28**		1.61**	–

CRIES = Children's Revised Impact of Event Scale; PTSD = posttraumatic stress disorder.

* *p <* 0.10;

** *p <* 0.05.

ROC curve analyses suggest that the five additional items that were significantly associated with anxiety disorders could differentiate well between children with and without an anxiety disorder (AUC=0.85, 95% CI: 0.81–0.89). A cut-off score of ≥10 emerged as the best balance between sensitivity (0.84) and specificity (0.73) for these five additional items associated with anxiety disorders. Performance statistics for this cut-off score and two other potential cut-offs are presented in Table 6. The six additional items that were associated with mood disorders demonstrated good discriminatory ability (AUC=0.85, 95% CI: 0.81–0.90). A cut-off score of ≥14 emerged as the best balance between sensitivity (0.82) and specificity (0.73). Performance statistics for this cut-off score and two other potential cut-offs are presented in Table 6. The additional items that were significantly associated with behavioral disorders had poor to fair discriminatory ability (AUC=0.72, 95% CI: 0.63–0.81). There was no clear cut-off point for these items found.

**Table 6:** Performance statistics for the additional items that were significantly associated with anxiety disorders and mood disorders

	Anxiety disorders			Mood disorders		
		
	Cut-off≥9	Cut-off≥10	Cut-off≥11	Cut-off≥13	Cut-off≥14	Cut-off≥15
Sensitivity	0.89	0.84	0.79	0.87	0.82	0.75
Specificity	0.68	0.73	0.78	0.70	0.73	0.77
PPV	0.57	0.59	0.63	0.32	0.34	0.35
NPV	0.93	0.90	0.89	0.97	0.96	0.95
Overall efficiency	0.74	0.76	0.78	0.72	0.75	0.77

Sensitivity is the proportion of children correctly identified as having a disorder; specificity is the proportion of children correctly identified as not having a disorder; PPV (positive predictive value) is the proportion of children who screen positive on the additional items and actually have a disorder; NPV (negative predictive value) is the proportion of children who screen negative on the additional items and do not have a disorder; overall efficiency is the proportion of children correctly identified by the screening tool.

## Discussion

The purpose of this study was to evaluate the screening accuracy of the CRIES-Plus, that is, the CRIES-13 in combination with several additional items, to detect PTSD and common comorbid disorders in children and adolescents exposed to various traumatic events. The results of this study indicate that although PTSD was the most prevalent disorder in the aftermath of traumatic events, children reported high levels of psychiatric comorbidity. Approximately 60% of children with PTSD had at least one comorbid disorder, confirming the high rate of comorbidity found in earlier studies of self-reported psychiatric disorders in children with PTSD (Copeland et al., 2007; Famularo et al., 1996; Suliman et al., 2009). Although all children in the study were exposed to at least one traumatic event during their lives, children with PTSD were more likely to show comorbid psychiatric disorders than children without PTSD. However, it cannot be ascertained whether the comorbid psychiatric disorders were the result of exposure to traumatic events or existed prior to exposure. In the latter case, it might be possible that children with a prior psychiatric diagnosis are more vulnerable to develop PTSD in the aftermath of a traumatic event.

Almost all additional items were individually related to more than one psychiatric disorder. This could possibly be explained by the substantial amount of symptom overlap between psychiatric diagnoses, particularly between PTSD, ADHD, depression, and anxiety disorders (Brady, Killeen, Brewerton, & Lucerini, 2000; Daud & Rydelius, 2009). Although these disorders have been regarded as conceptually distinct, research has demonstrated a considerable overlap in symptomatology (Antony & Stein, 2009). The results of this study also show that combinations of several additional items were strongly related with mood disorders, behavioral disorders, and anxiety disorders. More specifically, 6 out of 12 additional items (items 1, 3, 4, 7, 9, and 12) were significantly associated with mood disorders, 3 out of 12 items (items 6, 8, and 9) were associated with behavioral disorders, and 5 items (items 1, 3, 8, 9, and 11) were associated with anxiety disorders. Moreover, the additional items associated with mood disorders and anxiety disorders demonstrated good discriminatory ability, with a cut-off score of ≥14 and ≥10, respectively. The additional items that were associated with behavioral disorders had poor to fair discriminatory ability, with no clear cut-off point. Presumably, children are not reliable reporters of behavioral problems, confirming the general assumption that children might be better informants of internalizing problems, whereas parents are better reporters for externalizing disorders (Jensen et al., 1999). As expected, none of the additional items significantly improved the prediction of PTSD when added to the total scale of the CRIES-13. This may be due to the already excellent screening accuracy of the CRIES-13 itself (Verlinden et al., 2014b) and because PTSD was not the essence in formulating the additional items. Altogether, the ultimate CRIES-Plus consists of 13 items to screen for PTSD (the original CRIES-13) and eight additional items to screen for psychiatric comorbidity, that is, mood disorders (items 1, 3, 4, 7, 9, and 12) and anxiety disorders (items 1, 3, 8, 9, and 11).

Although our results indicate that the CRIES-Plus might be an effective tool to screen for PTSD and psychiatric comorbidity, we emphasize that screening tools should not be used as a replacement for a full assessment. If the CRIES-Plus indicates the presence of PTSD and/or psychiatric comorbidity, further clinical assessment is necessary in order to guide proper diagnosis and care. Other questionnaires to assess anxiety, depression, or behavioral symptoms can be helpful in these cases. Moreover, proper clinical diagnoses rely on a large amount of detailed information obtained from diagnostic interviews that assesses not only the severity of the symptoms, but also the duration and subjective impairment in academic, social, or other areas of functioning. As diagnostic interviews are time consuming and often require professional training, we recommend a stepped procedure, first screening children for PTSD and psychiatric comorbidity and then providing a more extensive assessment only to those children who screen positive. In this, the CRIES-Plus may serve as a first step to identify children who may need further assessment or treatment.

This study is limited by the fact that data were based on child self-reports. Although children are often quite aware of their internal state and feelings, parent reports are definitely necessary as a core part of comprehensive clinical assessment. Future research might address this limitation by examining the usefulness of the additional items on the parental version of the CRIES-13 (Verlinden et al., 2014a). Our study was further limited in that data were used from a sample with a reasonably high base-rate of PTSD. We therefore do not know how findings would generalize to populations with lower base-rates of PTSD. Further research is needed to find out whether similar results hold true in other populations.

Notwithstanding these limitations, the findings of this study provide support for the use of several additional items to screen for common psychiatric comorbidity in children with possible PTSD. However, further research into the reliability and validity (e.g., convergent and discriminant validity) of the CRIES-Plus is necessary before it can routinely be used in clinical practice. To conclude, a lot of controversy exists around the new DSM-5 and the proposed ICD-11 diagnostic criteria of PTSD with regard to the classification of comorbid symptoms. The proposed distinction between PTSD and complex PTSD in the ICD-11 has not been adopted in DSM-5. In spite of this, we would like to emphasize that the need to specify and target comorbid symptoms is very important, whether these symptoms are part of (complex) PTSD or not. In fact, better understanding of psychiatric comorbidity is highly relevant with respect to children's general health but may also help clinicians in assigning appropriate follow-up diagnostic and clinical care.
